# Health Inequalities in Crisis Times: Questions for Global Health Governance

**DOI:** 10.1177/27551938251381110

**Published:** 2025-10-16

**Authors:** Paul Crawshaw, Joanne Gray

**Affiliations:** 1 Social Sciences, Humanities and Law, 5462 Teesside University, Middlesbrough, UK; 2 Health and Life Sciences, 5995 Northumbria University, Newcastle upon Tyne, UK

**Keywords:** crisis, historical determinants, health governance, inequalities, syndemic

## Abstract

Health inequalities continue to blight populations creating a global social justice challenge. Despite continued action by policy makers and practitioners, they are recurrent and stubborn, with evidence of exacerbation in many territories. Driven by extractive, capitalist economic and political logics that have organised societies with systemic power imbalances, inequalities are so entrenched they appear ahistorical. Recent global crisis of disease, finance and conflict have intensified challenges, with outcomes worst for those with the least power and resources. Impacts are compounded where inequalities are structural and systemic, for example the result of institutionalised racism, unevenly distributed wealth and employment opportunities, lack of availability of suitable housing and food or limited access to education. Understanding health inequalities as dynamic, the outcome of complex ecologies and interactions as well as their historical antecedents and determinants is vital for public health research and practice. The differential impact of COVID-19 on populations has illuminated this, leading researchers to reframe the pandemic as syndemic. Such analysis is imperative both for immediate crisis response and future health protection planning and strategy. Our discussion highlights how historic and systemic inequalities shape contemporary population health, constructing stubborn barriers to social justice, an effect heightened during times of crisis like the COVID-19 syndemic. What is to be done to ameliorate their impact on the most vulnerable remains a vital question.

*What differentiates materialist epidemiology from bourgeois social epidemiology is the attempt to relate the patterns of disease and illness in a society to the economic and social relations which are the determinants of the functioning of that society.*

– Schnall and Kern^(1, p. 2)^

*If we fail to understand precaritiztion, then we understand neither the politics nor the economy of the present.*

– Lorey^(2, p. 1)^

The social sciences have a long tradition of engagement with historical methods to better understand the present and its antecedents, analyse the origins and emergence of norms, structures and systems and problematise contemporary social practices, values and beliefs. Scholars have used historical methodologies to establish causality, correlations and patterns that offer insights both for our present day and for what human futures might look like. From Wollstonecraft's *Vindication,*^3^ Marx's historical materialism,^4^ Popper's *Poverty of Historicism*^5^ to Foucault's^6,7^ genealogical investigation of systems of thought, social science has sought to understand the present, not only with empirical and theoretical interrogation of the contemporary social world, but through critical analysis of how historical antecedents assemble powerful and durable systems of control and order that structure the *now*, the temporal present in which our daily struggles are played out. From the hegemony of patriarchy to the dominance of capitalist political economies, systems that become *normal* profoundly shape the historical development of our societies; histories whose influence on our lived experience of the present cannot be overstated (Following Marx, ‘Men (sic) make their own history, but they do not make it as they please; they do not make it under self-selected circumstances, but under circumstances existing already, given and transmitted from the past. The tradition of all dead generations weighs like a nightmare on the brains of the living’.^(^^8^^, p. 5)^).

While it is uncontroversial to posit that historical antecedents shape contemporary social systems, cultures, and practices,^9^ less attention has been paid to the material legacy of structural inequalities; inequalities so deeply entrenched they are often misrecognised as ahistorical and immutable features of capitalist modernity. The consequences for those without power and resources are profound. In rich and poor nations alike, marginalised populations exist outwith the halo of progressive modernity, in a permanent present of social vulnerability and enduring precarity.^2^ Not only do the many remain poor,^10^ 14 million people in the United Kingdom,^11^ so will their children. As Todd's^12^ analysis of a century of U.K. mass observation data confirms, social mobility, the capacity to change socio-economic status by moving from one class to another, remains a niche phenomenon with profound consequences for the intergenerational reproduction of disadvantage. Grounded in the lived experience of the marginalised, Todd's historical sociology illustrates how faith in free markets as a route to equality and prosperity has been misplaced. The result has been the enduring reification of rigid social stratification across much of the twentieth century, punctuated by a brief ‘golden age’ in the 1960s. In this decade, the foundations of universal welfare and enhanced educational opportunities laid in the immediate post-war period delivered upward mobility in occupation and incomes as well as asset wealth through home ownership, providing for some a stake in society unknown to their parents. Progress was short lived. From 1979 onwards, Thatcherism and Reagonomics heralded rapid deindustrialisation, mass privatisation of public assets and the beginning of a neoliberal era^13^ that delivered dramatic increases in inequality across the high-income nations, reductions in living standards and an entrenchment of low social mobility.^12^

Whilst the marginalised languish in an unequal present, hegemonic, neoliberal visions of progressive modernity depict the acceleration of social goods such as improved living standards, access to new health technologies, safety, and security as universal, stable features of the rich nations. This unequal present is a direct outcome of historical forces that have organised societies with lasting legacies of structural power imbalances, the result of impositions such as extractive capitalist forms of production and systemic discriminatory racial stratification. The material effect is that, despite hegemonic visions of a progressive accelerated modernity, inequality is a persistent feature of societies globally.^14^ Health inequalities, those avoidable, unfair and systemic differences in health burdens and outcomes that disproportionately blight populations with the fewest resources^15^ are our concern here.

Given its mission of protecting and improving population health, the implications for public health of recurrent and stubborn inequalities in health outcomes are profound. Core to public health methodologies, epidemiology maps incidence and prevalence of health burdens, their causes and distribution at the population level, providing an evidence base for policy, planning and interventions. While populations are the empirical focus of inquiry, the individual does not disappear within these epidemiological imagined communities. Rather, it is at the level of the individual that the social production of health inequalities is played out. Following Krieger's^16^ ecosocial theory of disease distribution, ‘people literally biologically embody their societal and ecological context, at multiple levels, across the life course and historical generations’ (p. 636). The fundamental question becomes ‘who and what makes populations and their means?’ (p. 667). Our contention is that meaningful answers to this question must synthesise ecological and historical paradigms. When policy makers and practitioners confront social problems like the elevated health burdens faced by specific populations and their subgroups, their solutions are firmly located in the *present*.

What do we mean by *solutions are firmly located in the present?* Where else would solutions to chronic health burdens like noncommunicable diseases (NCDs) associated with, for example, poor diet or risky behaviours, be located other than the present, the *now* in which they are manifest? Our argument is that, although for sure policy makers and practitioners must posit solutions in the present day, the family failing (Here we paraphrase Nietzsche's contention that a lack of historical sense is the ‘family failing’ or ‘hereditary fault’ of all philosophers.^(^^17^^, p. 6)^) of public health interventions is to expect health to be improved, and inequalities reduced, by targeting individuals and populations in the here and now, with actions capable of impacting independently of, and unhindered by, broader historical and social processes and their legacies – factors that profoundly shape health experiences and expectations. Here, *static* views of health inequalities and strategies for amelioration fail to appreciate their *dynamic* nature, that they are the result of, for instance, long term political trends that have accepted low pay and unemployment as a feature of capitalist economies, normalisation of corporate systems that promote mass consumption of unhealthy food,^18^ or structural racism that segregates non-white populations into areas of environmental degradation.^19^ In the vocabulary of regional studies, they are pathway dependent^20^; historical structures powerfully determine present outcomes and future trajectories.

Our proposition is that strategies to tackle inequalities in the *present* must be cognisant of the historic and durable structures that determine the conditions of existence within which the micropolitics of health are played out.^21^ Such a present may, for example in the northeast of England where we are writing, be irrevocably shaped by rapid unmanaged deindustrialisation, commencing in the 1960s, resulting in persistent economic inactivity, unemployment, underemployment, and widespread poor work, all predictors of poor health outcomes. Five decades on, chaotic, unjust transitions continue to shape the health and life outcomes of these communities.^22^ Where contemporary public health strategies have oscillated between, at worst, behavioural, individualised solutions, to, at best, limited attempts to address environmental determinants, the problem has been approached as *static* and solvable through appeals to individual volition in pursuit of a transcendental model of good health,^23^ an ambition achievable for all with the right guidance, tools and motivations. The failure of this mode of health governance is writ large in the growth of inequalities; growth accelerated by health emergencies like COVID-19 that have disproportionate impact upon those populations with the least power and resources.^24^

In what follows we critically consider the persistence of health inequalities, the relationship between past and present and what this means for the prevalence of ill health in our most vulnerable communities. Health inequalities do not emerge in a vacuum. Rather, they are the outcome of unequal power relations and resource distribution that simultaneously determine both opportunities for good health and experience of ill health burdens. In turn, these relations are the product of historic social organisation and public policy formations that order the political economies of health within which inequalities manifest. We critically consider evidence that responses to COVID-19 that used the traditional tools of epidemiology to track, model, isolate and vaccinate, though effective in control and management as crisis response, were incapable of accounting for the wider structural and historical determinants that shaped the experiences of populations both in the present and into the future. Syndemic^25^ framing illuminates how the impacts of such health emergencies are significantly shaped by pre-existing stress points and vulnerabilities in societies. These drive complex interactions and determine the distribution of health burdens and outcomes with the already precarious and vulnerable most at risk. Understanding this is vital for preparedness for future emergencies, for tackling inequalities and ultimately the pursuit of social justice. To ignore is to condemn already disadvantaged populations to a permanent present of social vulnerability.

## Health Governance in Crisis Times

It is commonplace to characterise the early twenty-first century as a period of ongoing and accelerating crisis driven by confections of conflict and economic and political factors global in reach and effect.^26^ They include the financial crash of 2008 whose impacts continue in the austerity responses of governments, ongoing conflicts in the Democratic Republic of the Congo, Ukraine, Yemen and Sudan resulting in mass displacements of people, climate breakdown, the effects of which are most profoundly felt in the poorer nations, including enforced migration and the COVID-19 pandemic, which saw unprecedented economic and social disruption. As we write Israel's assaults on Gaza continue with devastating humanitarian consequences. Much could be added, but it is incontrovertible that the early years of the new millennium have brought geopolitical, economic, and social turbulence, arresting and reversing what many in the late twentieth century assumed to be the steady advancement of modernity and accompanying ontological security.^27,28^ The result has been described as a radical discontinuity of progressive globalisation.^29^

Amidst these crises transnational policy initiatives like the U.N. Sustainable Development Goals (SDG) struggle to deliver on ambitious plans to eradicate inequality and improve the lives of some of the world's most vulnerable populations.^30^ Simultaneously, in the WEIRD nations (Western, educated, industrialised, rich, democratic;^31^) neoliberal governments have prioritised market freedoms and capitalist expansion over social investment.^13^ The uncomfortable truth of our global present is that, despite boasts of growth, prosperity and convergence,^32^ in many nations, such advances have occurred in tandem with a devastating acceleration of socio-economic inequality^14^ and concomitant decline in social justice.^33^ This is not an historical accident. Rather, it is the outcome of political ideologies that gained hegemony in the second half of the twentieth century, driving unequal distribution of resources and wealth and ensuring marginalisation and precarity for many. As Storey^2^ argues, precarity is an organising principle of such hegemonic neoliberal models of economic and social organisation, the outcome of multiple forms of historic domination and exclusion, which, by regulating the minimum assurance of security, whilst proclaiming an absence of alternatives, becomes an instrument of government, whereby the productive individual must engage with endless self-management if they are to remain enfranchised. Here, health inequalities become a sine qua non of modern polities, predicated as they are upon individual management, and minimum assurance of, security from risks that are a direct outcome of capitalogenic environments.^34^ The imperative to be healthy^35^ becomes the mantra of health governance, with structural and systemic solutions sidelined in favour of the panacea of self-management.

## Health Inequalities as Persistent and Recurrent

Reducing inequality, or failure to do so, looms large in contemporary discussions of global governance. At the midpoint of a fifteen-year lifespan, progress in U.N. SDG 10, whose explicit aim is to ‘reduce inequality within and among countries,’ has been described as negligible.^30^ Concerns are framed in a broader interrogation of why, despite widespread acceptance of inequality as a global *bad*, the source of historic and recurrent immiseration of populations in both rich and poor nations, governments and intragovernmental organisations have failed to translate swathes of robust evidence into effective policy and action.

Failure is less surprising when examined through the lens of critical social science. Following Wilkinson and Pickett,^33^ addressing inequality is not only a moral imperative, demanding that those possessing power and resources must acknowledge that a social order where, by virtue of social position at birth, populations are condemned to poor life outcomes in the present and future, is reprehensible. For sure, reducing inequality is critical to improving the lives of billions of the most vulnerable globally, through providing more equal access to education, social infrastructure and welfare, the foundations of healthy communities. Simultaneously, inequality is bad for societies overall, leading to higher incidence of social problems, those behaviours and conditions that are perceived by the majority to be harmful, undesirable and in need of attention and resolution. These problems manifest in various forms including, poverty, crime, homelessness, substance abuse, violence, sex and racial discrimination, environmental degradation and unequal access to healthcare, education, and food. Wilkinson and Pickett's social epidemiology documents the correlations between inequality, social problems and welfare indicators across nations. Considering children's well-being, drug abuse, education, incarceration, mental and physical health, obesity, teenage pregnancies, trust and community life and violence, their analysis demonstrates that incidence and prevalence are higher in richer, but more unequal, countries, with negative outcomes disproportionally impacting those with the least resources. Their novel insight is that inequality itself leads to higher incidence of such undesirables throughout societies and at all levels, with deleterious social problems existing simultaneously as *cause* and *symptoms*. Consequently, unequal societies are not bad only for communities who bear the brunt of, for example, poverty or environmental degradation (and thus those populations *not* impacted should have a moral imperative to help), but also for those further up the social gradient and in possession of the power, resources and the security they bring. Greater inequality means greater precarity and insecurity for all.^33^

Simultaneously there remains a lack of consensus on how health inequalities should be addressed, and, where robust evidence is available, there is a stubborn research-to-implementation gap.^36^ It is not that interventions do not exist, in fact they proliferate.^37^ Yet too often efforts are diverted to proximal, behavioural factors like tobacco use or poor diet. These behaviours, already clustered within populations of lower socio-economic status, are symptomatic of the stresses and systemic limiting of opportunities, themselves the results of inequality; the so-called causes of the causes.^38^ Reflecting a troubling lifestyle drift,^39^ they eschew challenges to distal structural and systemic determinants in favour of strategies that manage populations and shape behaviours through new forms of health governmentality.^7^ Positing an idealised, universal subject willing and able to manage their own health, these governmentalities are biopolitical, exercising power over bodies through the regulation of life itself.^40^

Though ostensibly predicated upon promoting better population health management, such doxa risk producing naturalised and dehistoricised notions of life,^41^ simultaneously abjuring the disruption of historic economic and social systems that sustain inequality. Paradoxically, they espouse better community and individual level responses to health challenges, even where they are the transparent outcome of ecosocial issues – for example, entrenched degradation in urban environments disproportionately populated by low incomes and minority groups.^19^

Where classical public health sought to modify social, economic, and environmental factors through systemic and structural interventions, these biopolitical methodologies focus upon influencing and improving lifestyles and behaviours *at a distance*.^42^ This transition from a health governance underpinned by social engineering to one predicated upon techniques and strategies to shape behaviour and manage populations is an epochal one. Politically aligned with the neoliberal consensus, newer forms of health governmentality are congruent with market freedoms, individual agency, and the enterprising self.^7^ The outcome of this shift is a form of health governance that remains firmly at a distance, eschewing direct intervention in favour of a biopolitics of population management to be achieved through information, education, and the judicious use of technologies that shape individual behaviours and choices. As Esposito^(^^41^^, p. 87–88)^ describes, these methods risk ‘overlooking equally important topics related to modes of production, class struggle, and constituent and constituted powers’. By adopting universal governance models that assume health improvement can meaningfully operate at the level of individual behaviours and choices, they posit a kind of ‘pan-biopoliticism’ that flattens historical specificities into a single biological mold. Here, life, deprived of historical context, would remain imprisoned in a biological cage that cancels out the rhythm of existence’.^(^^41^ ^p. 87–88)^ The material result is that we lose sight of how different societies work in the concreteness of their processes, including the historical determinants of inequality. In contrast, a more dynamic perspective illuminates how good health is unlikely to be the outcome of individual behaviours and choices made in stable and static settings where individual volition is sovereign. Rather, it is over determined by our position in dominant economic and social ecosystems, themselves shaped by dynamic but durable power structures that are unequal by design. The outcome for many is curtailed access to opportunity and resources, resulting in persistent marginalisation and precarity.^2^

Foucault's historical epistemology provides a foundational framework for interrogating these histories of the present.^43^ As Esposito notes, ‘The peculiar object of Foucault's study is not facts or concepts themselves, but the condition of their historical emergence, the space–time context that leads to their particular configuration’.^(^^41^^, p. 89)^ He allows us to appreciate how constituting processes, like health governance, are not only profoundly shaped by historical determinants, but that simultaneously the practices they promote are limited by ecosocial antecedents, themselves constrained by the contexts in which they are located. We are not condemned to incarceration in history, however. If we adopt a critical standpoint, located in the present, but cognisant of the systemic organisation of oppression that is the reality for marginalised populations, we may always be located in a historical constellation, but it is one that we can modify by creating friction with our conditions of existence.^41^ Adopting this position, and the possibility of disruption it presents is of course far more likely to be the luxury of the economically secure and educationally privileged as a condition of their existence. The less advantaged remain condemned to a permanent *present* of social vulnerability, a condition of precarity shaped by historical oppression and a manifestation of path dependency that recurrent and stubborn marginalisation renders them powerless to challenge.^2^

Modernist conceptions of health as a status obtainable through judicious management of prevention and self-actualisation are, of course, epistemically hegemonic, centring as they do the experience of WEIRD nations. In the Global South of low- and middle-income countries, territories that contain 85 percent of the world's population, account for 39 percent of its gross domestic product (GDP;^44^), and which through decades of unequal exchange and extraction, contribute trillions to the economies of the North,^45^ health burdens are still driven by a double bind of both noncommunicable and infectious diseases. This dissonance is reflected in global health governance: ‘The World Health Organisation (WHO) strives to conceive health and health risks in a universal manner applicable to all human beings. This highly commendable desire presumes that life is the same everywhere. However, life in the countries of the North is taken as the reference and its pertinence to the poor countries is debateable’.^(^^23^^, p. 1)^.

Examining malaria in Benin, Nigeria, Lysaniuk and Tabeaud^23^ document how vulnerability to health risks goes far beyond exposure to a pathogenic agent. Rather, health is the outcome of interacting systems at different spatial and temporal scales and deterioration in health the result of the impact of environmental, social and economic determinants, or combinations thereof, which make certain individuals or groups vulnerable. Precedent profoundly shapes these vulnerabilities. It is no accident that these communities were previously colonised, with impacts felt to the present day, and into the future. These impacts impair development, sap human and natural resources and leave legacies of poverty, insecurity, and conflict.^46^ As Piketty^47^ states, rich countries’ prosperity would not exist without the poor countries. The growth of wealth in the Western world has long been based on the international division of labour and the feverish exploitation of natural and human resources worldwide. These legacies continue to shape the lives of millions globally.

## Socio-Economic Determinants in Weird Nations

The persistence of inequality in WEIRD nations, despite their evident prosperity,^(^^48^ ^p. 761)^ is not anomalous but indicative of the structural logics underpinning contemporary global capitalism. For Mackenbach^48^ the coexistence of prosperity and inequality represents a failure for public policy, which, throughout the twentieth century has aimed for continuous advancement, in line with liberal progressive visions of social improvement and the amelioration of *bads* associated with earlier phases of modernity (incidence and prevalence of infectious disease; high child mortality, and so on). Here, failure is the outcome of a lack of ambition in redistributive policies that serves to entrench inequalities in health and consumption behaviours that impact more negatively on lower socio-economic strata (p. 767). The latter, Mackenbach posits, is a result of the temporal drag that exists between different socio-economic groups and their ability to desist or reduce unhealthy behaviours known to be proximal determinants of both conditions associated with health risks, for example, consuming an excess of fatty foods and obesity, and their concomitant complications. These behavioural determinants are simultaneously shaped by both individual and structural factors as those with greater wherewithal are more likely to adopt healthy lifestyles. In sociological terms, structure determines agency.^49^ The material outcome are class habitus (Following Bourdieu^49^, *habitus* is a system of ingrained dispositions shaped by social conditions that guide how people think, feel, and act.) defined by stubborn inequalities, marginalisation and precarity.

These dynamics have profound implications for health governance in WEIRD nations, necessitating a reorientation of policy paradigms to effectively address entrenched inequalities. Though commentators may posit the convergence of health and social policy as a more recent phenomenon,^50^ the history of public health in the Global North is of a movement committed to improving the living conditions of working people through the organised efforts of society, often in direct response to dramatic and rapid social change. In eighteenth-century England, for instance, punitive land enclosures and rapid industrialisation drove populations into urban environments. Emerging towns and cities characterised by poverty and squalor exacerbated by a lack of infrastructure such as adequate sanitation and their newly immiserated populations became the target of earnest reforms determined to improve environments and physical health.^51^ Reformers recognised that although the science of public health may be closely aligned with biomedicine, using epidemiological methods to map mechanisms of infectious diseases, pathogens, pathologies and patterns of distribution within populations, its priorities, actions and practices must have more in common with those of the housing officer, social worker, schoolteacher and town planner.^52^ Effective governance must straddle a range of societal institutions, delivering health in all policies, recognising its ecological drivers and that outcomes will only be improved for populations through purposeful reshaping of the environments in which people find themselves and that structure opportunities to be healthy. The limitations of a flat, pan-historical biopolitics, one that abstracts health governance from its socio-political contexts, are clear.^42^

## Covid-19 was Not a Pandemic

The rapid emergence and spread of the novel COVID-19 virus in early 2020 created a global health emergency for which few countries were prepared. Despite claims of being a great leveller and a resulting democratisation of risk, the effects of the pandemic were felt variably, and most significantly, unequally^23^ within populations. As Horton posits, ‘Two categories of disease are interacting within specific populations – infection with severe acute respiratory syndrome coronavirus 2 (SARS-CoV-2) and an array of NCDs. These conditions are clustering within social groups according to patterns of inequality deeply embedded in our societies. The aggregation of these diseases on a background of social and economic disparity exacerbates the adverse effects of each separate disease. COVID-19 is not a pandemic. It is a syndemic’.^(^^53^^, p. 874)^

*Syndemic*, a portmanteau of synergy and epidemic, captures how health emergencies like COVID-19 impact populations in highly unequal ways, deepening existing social fractures and disproportionately affecting the already marginalised. Horton's syndemic framing finds a trenchant complement in Davis's^54,55^ work, which exposes how systemic neglect and capitalist exploitation of environments actively produce the uneven health burdens that crises like COVID-19 expose. Davis's prescient intervention of the early 2000s^54^ and post COVID-19 revision^55^ has highlighted the capitolegenic effects of environmental degradation, chaotic urbanisation and factory farming on human health. Here, political economies determine not only how health crises are managed, but how they produce the adverse conditions that make crisis inevitable. The outcome is that global capitalism not only accelerates the emergence of zoonotic disease but ensures disproportionate impact on the most vulnerable through austerity, inequality and racialised abandonment.^54^ Public health crises are both materially and structurally produced by capitalist political economies. As Davis^54,55^ and Horton^53^ predicted, during COVID-19 the toll was not only lives lost as a direct result of the virus, but the long-term economic distress that responses and their aftermath would bring to already marginalised communities – communities that bore the brunt of morbidity and mortality as variations continued to spread into 2021. Simultaneously, pre-existing inequalities rendered the marginalised disproportionately vulnerable. This differential impact of COVID-19 on social groups has illuminated how a priori risk and vulnerability continue to be powerful determinants of incidence, prevalence, and experience of disease.

As Davis and Horton anticipated, COVID-19 not only claimed lives directly, but also deepened long term economic and social distress among already marginalised communities. Empirical studies support these contentions. Analysis of 3,141 U.S. counties found that, in 2020, the most disadvantaged (measured by the Social Vulnerability Index [SVI]) had on average twice the rate of COVID-19 cases and deaths relative to the least disadvantaged.^(^^56^^, p. 499)^ Incidence of chronic disease was also substantially higher in disadvantaged counties. Islam and colleagues^56^ conclude that some experienced a confluence of epidemics from COVID-19 and chronic disease, where each exacerbates the other and health inequalities widen: a paradigm of Horton's syndemic.

Such material effects were not temporally limited to the duration of the health emergency. Long COVID-19, now widely recognised as a clinical phenomenon with significant impact on sufferers’ daily activities with symptoms such as fatigue, persistent cough, breathlessness, and loss of concentration^57^ continues to be a health burden. Not only are the socially vulnerable more likely to experience long COVID-19, but they also experience significant barriers to accessing support services.^(^^58^^, p. 10)^ The outcome is entrenchment of health burdens and inequalities within vulnerable populations. Prashar^58^ has similarly pointed to the necessity of providing community-based services if equitable levels of care are to be achieved for those already vulnerable groups who are at significantly higher risk of life limiting impacts from long COVID-19. The implications for the future health of already historically vulnerable social groups are clear.

## Historic Health Inequalities and the De-Democratisation of Risk

Sociologist Ulrich Beck^59^ famously posited that in modern industrial societies, ‘risk is inversely distributed in proportion to wealth’ (p. 19); wealth accumulates at the top, risk at the bottom. Here, Beck did not merely tap into the anxieties and insecurities that characterise late capitalism. His work inculcated an understanding that the ‘major threats that societies face are no longer primarily external, coming from without – most obviously as natural hazards. Rather, they are produced as unintended consequences of modernisation itself’.^(^^59^^, p. 20)^ Simultaneously, they are global in impact. We cannot hide from the deleterious consequences of climate breakdown, diminishing antibiotic resistance or socio-economic vulnerabilities created by extractive systems of production and unequal distribution of resources, as witnessed when COVID-19 disrupted public life, restricting education, access to spaces and shutting down the economy. Much has been written about COVID-19 as an inevitable outcome of mass animal agriculture, global travel, and extractive food production with demands that lessons be learnt for future health governance. If policy makers are serious about ameliorating future harms, they must simultaneously understand how interaction with enduring, sedimented, inequalities dictate that both exposure to risk and negative outcomes are not democratic.^60^ Rather, they are profoundly determined by historic, pre-existing inequalities and systemic disadvantage that shape future health burdens for individuals and communities alike.

*What do we mean by this?* When crises emerge and disease interacts with pre-existing vulnerabilities, some social groups are impacted more profoundly than others.^24^ Entrenched and recurrent inequalities position populations in a permanent state of social vulnerability, powerfully shaping their present and futures through curtailing social mobility and embedding intergenerational disadvantage.^12^ As Calestani^61^ has observed in southern Italy, mechanisms may act in individual lifeworld's, however they are subsumed within more complex historical, economic, and environmental processes as marginalised populations grapple in the present with the toxic legacies of the past. Approaching these issues as anthropogenic, originating in human activity and as political and socio-economic problems, symptoms of global inequalities and injustices, Calestani defines the Anthropocene as a ‘sociogenic’ phenomenon emerging from uneven distribution of power and social relations (p. 1). Such analysis opens the possibility of a radical critique of capitalist and (post) colonial relations that operate to determine unequal access to resources for the poor, and power relations that are shaped by the past, but which determine human futures in profound ways.

## The Political Economy of Health or Historical Materialism Bites Back

Despite hegemonic visions of progressive and inclusive modernity, inequality continues to be a wicked issue in the high-income nations.^62^ The United States, the world's largest economy, faces epidemics of opioid use directly correlated with low-income neighbourhoods and limited access to healthcare as well as entrenched racial discrimination that manifests in multiple ways, for example, with black and brown communities disproportionately the victims of crime and simultaneously the most likely to be incarcerated.^63^ In low- and middle-income countries, despite claims of convergence^32^ disproportionate numbers experience extreme, absolute poverty, lacking food security and with limited access to resources and infrastructure both to meet immediate needs like clean water and sanitation, as well as those such as education that allow communities to flourish and thrive. These issues are further exacerbated across regions by political instability and conflict. This is not the prosperous globalisation promised by late twentieth century proponents of a global neoliberal consensus.

Much has been written about the hegemony of a neoliberal global order that emerged in the second half of the twentieth century, with Fukuyama famously heralding the *end of history*, or at least the end of actually existing alternatives to a liberal democratic, capitalistic order. This assumed hegemony fundamentally reshaped economic and political systems with profound effects for nations whose leaders adopted it as an organising principle for governance and a pathway to prosperity for all, an economic theory that has been consistently discredited.^64^ Elsewhere, nations found themselves at the mercy of global systems driven by a form of capitalism whose extractive potential rapidly proved unprecedented with devastating impacts for low- and middle-income countries both in terms of conflict, disasters, economic insecurity and poverty.^45^ These rentier forms closely parallel colonialism, with the latter providing the foundations for capitals subjugation of first nation communities, stripping them of resources for the benefit of others.^65^ The extractive economic systems that emerged supplanted the autonomy of indigenous populations, replacing it with forced labour, compulsory cash crop production and delegation of power to trading companies, with resulting capital and wealth accumulation accelerating development in the Global North whilst spreading underdevelopment in the South.^66^ As Quinlan^67^ posits, effective redress requires global shifts towards sustainability and equality and reciprocity in terms of wealth and resources, all anathema to the discourse of rich ruling elites (p. 461).

The promise of an end to ideological conflicts between capitalism and communism, epitomised in the work of liberals like Fukuyama,^28^ heralding a peaceful globalisation and delivering prosperity for all, was always, at best, a counter intuitively optimistic aspiration. Hegemonic in nature, this teleological model had little foundation in the reality of geopolitical structures and struggles with a surprising absence of consideration of diverse lived experiences of significant proportions of the planet. The colonial and imperial antecedents that determined a fragile world order have cast a long shadow, a fact stressed throughout development, black and critical race studies,^46^ but only more recently in global studies of inequality. Post 2008, analysis, interpretation and representations of inequality in the western nations have come sharply back into focus.^14^ Following the financial crisis, governments, notably in the United States and United Kingdom, unleashed austerity programmes that rapidly exacerbated inequalities. A decade of immiseration followed, with particular impact upon the disabled, ethnic minorities, women, and children. The combination of ongoing conflicts that continue to destabilise regions and displace populations globally, creating economic, social, and political tensions centred on the movement of populations and the catastrophic rupture of the COVID-19 syndemic has for many, created a permanent crisis state. This stasis of crisis is far removed from the late twentieth-century promise of prosperity and a share for all in the spoils of a peaceful globalisation.

## Implications for Inequalities in Health

Within the WEIRD nations, the response to an increased understanding of health inequalities, their determinants and impact have fluctuated between the person-centred and behavioural and more environmental, ecological approaches. The political imperatives for this are not well hidden, with the former reflecting prevailing social and economic orthodoxies in which they are produced.^(^^68^^, p. 294–5)^

Over the past four decades these polities have been characterised by decollectivisation of welfare and a shift to neoliberalism as a mode of governance. Such economic and political organisation of liberal democracies has had significant implications for both understandings of, and policy responses to, health inequalities. Here, health citizenship is characterised as a relationship between individuals and the state that carries with it rights and responsibilities. Under what became known as the *new public health*, the notion of a healthy citizen emerged as an individual with a right to health^(^^69^^, p. 64)^ but, most significantly, as the bearer of personal responsibility to adopt the imperatives of the state and other agencies concerning the maintenance and protection of good health.^69^ Healthy citizenship became not merely the right to good health, something that will be provided by the state, but a duty to stay well, incorporating an understanding of health as a domain of individual appropriation through rational choice. Health must be played out as a series of duties and obligations, as an ethical imperative that the good citizen must work to achieve.^69^ In this way, individuals are expected to choose health-promoting lifestyles and exhibit healthy behaviours as part of the duty of modern citizenship.

Such late capitalist conceptions of a new public health that governs at a distance^42^ represents a radical rupture from historic models characterised by collectivity and social engineering. Adopting a neoliberal mentality that posits the individual as responsible for the management of their own well-being, they embody new political economies of health that mirror capitalistic forms of social organisation. These modes of governance fail to account for the wider determinants of health, the complex and relational contexts within which health behaviours take place, or the powerful historical drivers that shapes social relations in the present and in turn individuals’ ability to manage their own well-being. Revisiting Esposito,^41^ where we acquiesce to these imperatives, we run the risk of accepting a pan-biopoliticism that overlooks modes of production, class struggle, and constituent and constituted powers: the most powerful factors shaping the lifeworld's of the precarious and vulnerable (p. 88).

## What is to be Done?

Health inequalities research and scholarship have run a parallel, but not always integrated, approach with wider concerns with economic inequality that continue to exercise academics and activists globally.^14^ In WEIRD nations, public health remains anchored in individualised, lifestyle-oriented strategies for managing NCDs, emphasising behavioural risk and responsibilising individuals, while paradoxically invoking the language of social determinants and the essential ecology of human well-being, thereby deflecting attention from the structural conditions that produce and perpetuate illness.

Harvey^13^ proposes that neoliberal doxa has imposed a powerful new mental conception of the world (p. 131) that frames all human activity within the terms of reference of market logics, promoting individualism and an understanding of social relationships based on competition, imagining enterprising subjects, willing and able to construct a healthy self-identity under the guidance of distant expert discourses.^42^ These outcomes are not dictated by chance. Rather they are a direct result of differential social conditions and access to health protection as well as chronic failures of public policy.^48^

Revisiting COVID-19, it is clear that the outcomes of the syndemic represent not a rupture^70^ but rather a continuity both in how populations are differentially impacted by health crises and how they are exacerbated by inadequate policy responses that fail to address entrenched structural factors, factors that profoundly determine the pre-existing incidence and prevalence of disease in the societies in which health emergencies occur. Rather, the impact of the syndemic, in terms of prevalence, morbidity and mortality reflects deeply ingrained, historically determined inequalities in experiences of health and illness, exposing the vulnerability of populations faced with the perennial challenges of inadequate housing, poor work and social insecurity. Put simply, a priori risk and vulnerability remain the most powerful determinant of present and future health expectations and outcomes. The implications for intergenerational reproduction of inequality are clear.

Singer's syndemic state^25^ is not confined to moments of crisis but manifests as a persistent condition for communities facing structural disadvantage, shaping everyday experiences of health, housing, education, and compounded by systemic forces such as institutional racism.^19^ It exposes broader failures in public policy that have overdetermined populations’ differential exposure to risk through governance frameworks ill-equipped to address systemic inequalities, privileging instead a rhetoric of equality of opportunity, often exemplified by the provision of universal access to health and education within ostensibly meritocratic societies. The narrative of risk socialisation suggests that public policy protections, through welfare, education, and similar institutions, guarantee equality of opportunity within ostensibly meritocratic liberal democracies, enabling individuals and communities from diverse backgrounds to thrive, unimpeded by structural or institutionalised disadvantage. This standpoint is challenged by a weight of evidence from social science research that has consistently demonstrated how socio-economic status, ethnicity, gender, and location are key determinants of health experiences as well as wider life chances.^71,33^ Simultaneously corporate powers with greater global reach than governments or intragovernmental organisations alike ensure that commercial determinants of health^18^ continue to negatively impact lived environments and drive unhealthy behaviours. These impact disproportionally on lower socio-economic groups, for example, through inflated prevalence of obesity and its concomitant risks.^72^

How different social, economic, and political contexts hinder or enable the co-occurrence of health burdens must be considered ecologically and historically, demanding a dynamic understanding of the persistence and growth of inequalities within and between nations and their long-term impacts. Notwithstanding ongoing debates regarding convergence,^32^ there is clear evidence that the latter has systematically worsened in monetary terms since 1950.^73^ By 2022 the GDP per capita of the Global North was 12 times greater than that of the Global South, a ratio twice as big as in 1950, contradicting neoclassical endogenous convergence models that confidently posited an elimination of disparities as the countries of the South transitioned to full capitalist status.^(^^73^^, p. 3–5)^ The material outcome is a multipolar world divided into great geoeconomic blocs of which one is between 10 and 40 times wealthier than another.^(^^73^^, p. 33–34)^ For policy makers this demands a renewed vigour in embedding the goal of equity into all policies, prioritising human well-being and challenging the systemic disadvantage that ensures negative outcomes for the most vulnerable. Despite criticism of progress to date, the U.N. SDGs represent a bold attempt to unify ambitions to holistically address the cross-cutting issues of health, climate change and environmental degradation, conflict, food security, equity in access to education, gender inequality, racial discrimination, amongst many others, that collectively negatively impact the well-being of populations globally. Such attempts at meta-governance across nations are vital if policymakers are serious about tackling the challenges that so disproportionately affect the most vulnerable. To succeed, however, they must appreciate the significance of local contexts, and their origins, and be capable of influencing those able to shape them in ways that are beneficial to communities through prioritising human need and well-being over other interests. The challenges are clear.

## Conclusion

Thirty years on, Fukuyama's now (in)famous analysis that the decline of global power blocks in the late 1980s represented an epochal shift from a world characterised by conflict and tension between a capitalist west and communist east represented the end of history, the victory of liberal democracy and neoliberal economics and entry to a globalised, more peaceful and prosperous world, rings hollow. His eschatological vision proposed an endpoint in which, while nation-states would retain distinct identities, their territories would be unified through more equitable material wealth, shared democratic values, and reduced conflict, imagining a form of peaceful globalisation that would lead to greater egalitarianism. These ideas offer little serious consideration of the systemic limitations that have sustained the uneven development of structures and the formation of capitalist political economies. There is a poverty of historicism in this teleological vision of an epochal shift toward a stable world order that has consistently failed to materialise.^29^

Amidst these ongoing crises, eradicating health inequalities continues to be an urgent priority for practitioners and policy makers globally and the subject of a continually expanding field of academic research. Ezell^74^ has characterised the latter as a ‘health disparities research industrial complex’. Our intention with this article is not to add to this complex and simultaneously succumb to the risks of being ‘transfixed by a ceaseless, vague and colonialist pursuit of scientific knowledge, irrespective of potential utility, translatability, impact’.^(^^74^^, p. 6)^ Rather, in solidarity with Ezell we seek to disrupt technocratic solutions that characterise neoliberal health governance, solutions premised on a fallacious conception of the unhealthy individual as a static entity, capable of change in isolation from the systemic disadvantage and historical marginalisation that shape life within precarious societies.^2^ Following Davis,^54,55^ this requires critical analysis of the capitologenic determinants of health and health emergencies, analysis capable of illuminating how economic systems not only engineer social conditions, but amplify the consequences through systemic inequality, border regimes, environmental exploitation and the erosion of infrastructure. Davis underscores how, by prioritising profit over public welfare, capitalist political economies create environments where health crises are not mere accidents but inevitable outcomes of systemic structural inequality.

Despite epistemic hegemonies of technological acceleration and economic growth, deeply ingrained systemic inequalities continue to shape the health expectations of billions globally.^75^ Understanding the historical antecedents of these systems is vital if we are serious about reducing health inequalities in the present and future. COVID-19 highlighted that responses to health emergencies rely heavily on short-term measures such as lockdowns, testing, and rapid vaccine distribution. Once the pandemic was deemed endemic, however, emergency interventions quickly receded. In the aftermath, despite the clear impact of entrenched inequalities on health outcomes, public policy has done little to address the structural determinants of health and well-being. Rather, in failing to confront systemic issues like underfunded health systems, neoliberal policies, and growing economic disparities, political discourse has largely ignored the role of inequality in shaping outcomes, offering few long-term solutions. This oversight has significant implications for future health emergencies, undermining preparedness and response effectiveness. To continue in this way is to position the marginalised in a permanent *present* of social vulnerability, condemned only to weather, in a state of unpreparedness, the next crisis or health emergency. Returning to Krieger,^16^ what is required is an ecosocial standpoint that demands appreciation of the impact of inequalities across the life course and historical generations (p. 636). COVID-19 has exposed how we must understand the weight of historically determined structural and systemic inequalities in shaping the present and future health burdens faced by communities. Such an approach means challenging deeply ingrained inequities in the distribution of opportunities and resources that allow communities to be healthy. This approach cannot be predicated on an assumed universal human experience of health.^23^ Rather, it must recognise the many different determinants, current and historic, systemic, and economic, that shape populations’ health experiences and expectations and their ability to stay well. If poor health is the outcome of the intersection of historic forces, material circumstances and systemic disadvantage, it is the role of progressive public policy to build supportive ecosystems with the longevity to ensure good health for future populations. Our challenge is not merely for health, but for global, intergenerational social justice.
